# Prenatal undernutrition disrupted the sexual maturation, but not the sexual behavior, in male rats

**DOI:** 10.1002/rmb2.12045

**Published:** 2017-09-16

**Authors:** Toshiya Matsuzaki, Munkhsaikhan Munkhzaya, Altankhuu Tungalagsuvd, Yiliyasi Mayila, Takeshi Iwasa, Kiyohito Yano, Rie Yanagihara, Takako Tokui, Takeshi Kato, Akira Kuwahara, Sumika Matsui, Minoru Irahara

**Affiliations:** ^1^ Department of Obstetrics and Gynecology Graduate School of Biomedical Sciences Tokushima University Tokushima Japan

**Keywords:** prenatal undernutrition, preputial separation, sexual behavior, sexual maturation, stress

## Abstract

**Purpose:**

Exposure to various stressors, including psychological, metabolic, and immune, in the perinatal period induces long‐lasting effects in physiological function and increase the risk of metabolic disorders in later life. In the present study, sexual maturation and sexual behavior were assessed in prenatally undernourished mature male rats.

**Methods:**

All the pregnant rats were divided into the maternal normal nutrition (mNN) group and the maternal undernutrition (mUN) group. The mUN mothers received 50% of the amount of the daily food intake of the mNN mothers. Preputial separation and sexual behavior were observed in randomly selected pups of the mNN and mUN groups.

**Results:**

The body weight of the mothers was significantly lighter in the mUN group than in the mNN group. Similarly, the pups in the mUN group showed a significantly lower body weight than those in the mNN group from postnatal day (PND) 1 to PND 15. The preputial separation day was significantly delayed in the mUN group, compared to the mNN group. Sexual behavior did not show any significant difference between the two groups.

**Conclusion:**

These findings indicated that prenatal undernutrition delayed sexual maturation, but did not suppress sexual behavior, in mature male rats.

## 1 INTRODUCTION

The prenatal and early neonatal periods are important in brain development because neurons mature during these periods.^1^ Exposure to various stressors, including psychologic, metabolic, and immune, during these periods induces long‐lasting effects in physiological function and increases the risk of metabolic disorders in later life. The long‐lasting effects of stressors in the prenatal period have been widely studied in humans. Prenatal undernutrition‐induced pathogenesis of diseases, including hypertension, cardiovascular diseases, insulin resistance, and type 2 diabetes, in adulthood has been named as the origin of health and disease or metabolic programming.^2^, ^3^

It also has been reported that prenatal undernutrition has impaired the onset of puberty and the estrous cycle in male and female rats^4^, ^5^, ^6^ and has induced metabolic disorders.^7^, ^8^ Furthermore, the exposure to lipopolysaccharide (LPS) in the early neonatal period has delayed vaginal opening and preputial separation (PS) and disrupted sexual behavior in male and female rats.^9^, ^10^ However, it has been poorly investigated whether prenatal undernutrition influence sexual behavior or not.

The aim of this study was to investigate the effect of prenatal undernutrition on the sexual behavior of male rats in adulthood. In this study, PS was assessed as a reliable finding of the onset of puberty and sexual behavior of prenatally undernourished mature male rats.

## 2 MATERIALS AND METHODS

### 2.1 Animals

Eight pregnant Sprague–Dawley rats were purchased (Charles River Japan, Inc., Tokyo, Japan) and housed individually under controlled lighting (14 hours light/10 hours dark cycle) and temperature (24°C) conditions. All the animal experiments were conducted in accordance with the ethical standards of the institutional Animal Care and Use Committee of the University of Tokushima, Tokushima, Japan. All the pregnant rats were divided into a maternal normal nutrition (mNN) group and a maternal undernutrition (mUN) group. The mUN mothers received 50% of the amount of the daily food intake of the mNN mothers from gestation day 13‐21.^7^ After birth, the mothers were allowed ad libitum access to water and food during the lactation period. The body weight of the pregnant rats and pups was measured from gestation days 14‐21 and from postnatal day (PND) 1 to PND 35, respectively.

### 2.2 Sexual maturation and sexual behavior

Preputial separation is the separation of the prepuce from the glans and was examined from PND 28‐41 in all the pups.^11^ Fourteen mature female partner rats were prepared and used for testing the sexual behavior of the male rats. All the female rats were bilaterally ovariectomized at 8 weeks of age under sodium pentobarbital (40‐50 mg/kg; intraperitoneal)‐induced anesthesia. At 2 weeks after the ovariectomy, the rats were subcutaneously injected with estradiol benzoate (10 μg in 0.1 mL peanut oil; Sigma, St. Louis, MO, USA), followed 48 hours later with progesterone (1 mg in 0.1 mL peanut oil; Sigma). The sexual behavior was checked 4 hours after the progesterone injection.^12^, ^13^ The sexual behavior of the male rats was tested at 11 weeks of age under the following condition: an experimental male rat and a partner female rat were placed one‐by‐one in an acrylic cage (70 cm × 45 cm × 34 cm). After a 5 minutes habituation period in a cage together, the sexual behavior was monitored and recorded for 30 minutes. The copulatory sexual behavior, including the number of mounts (climbing onto the back of the female rat), intromissions (mounts followed by penile erection and insertion of the penis into the vagina), and ejaculations (mounts and intromissions followed by a deep forward thrust), were measured from each video recording.^14^

### 2.3 Statistical analysis

The difference in the body weight, PS day, and sexual behavior were analyzed by using Student's *t* test, Welch's *t* test, and Mann‐Whitney's *U* test after considering the variance and distribution. All the data are presented as the mean±standard error. *P*<.05 and *P*<.01 were considered to be significant in all the analyses.

## 3 RESULTS

**Figure 1:**
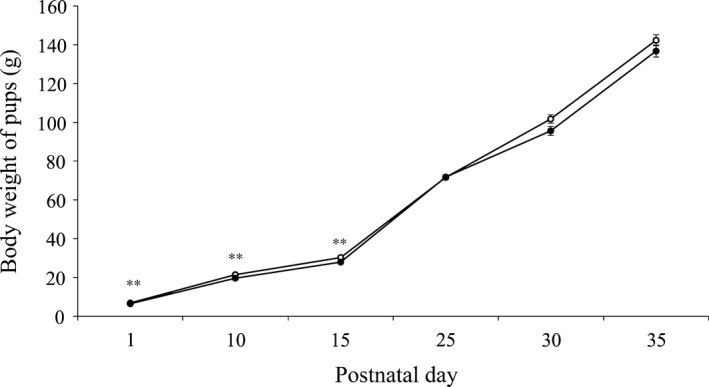
Body weight of the pups. The maternal undernutrition (mUN) (●) pups showed catch‐up growth during the neonatal period. In the mUN group, the body weight was significantly lower than in the maternal normal nutrition (mNN) group (○) from postnatal day (PND) 1 to PND 15. The data are presented as the mean±standard error. **P<.01 compared to mNN

The body weight of the mothers was similar on gestation day 14 between the mNN and mUN groups (mNN: 332.43±6.62 g; mUN: 334.45±4.61 g). The mothers of the mUN group showed a significantly lighter body weight on gestation day 21 (339.9±3.72 g), compared to the mothers in the mNN group (425.45±9.56 g) (*P*<.05). The pups of the mUN group had a significantly lighter body weight on PND 1, compared to the pups of the mNN group, and the difference persisted before PND 25, when the pups of the mUN rats showed catch‐up growth (Figure 1).

**Figure 2:**
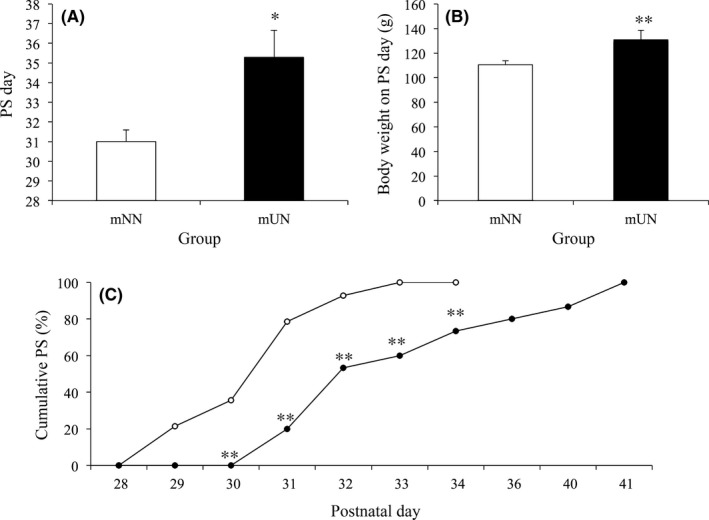
Effect of prenatal undernutrition on sexual maturation. The preputial separation (PS) day was later (A) and the body weight (B) on the PS day was heaver in the maternal undernutrition (mUN) group than in the maternal normal nutrition (mNN) group. The cumulative PS date showed a significant difference from postnatal day (PND) 30 to PND 34 between the mUN group (●) and the mNN group (○) (C). The data are presented as the mean + standard error. *P<.05 and **P<.01 compared to mNN

**Figure 3:**
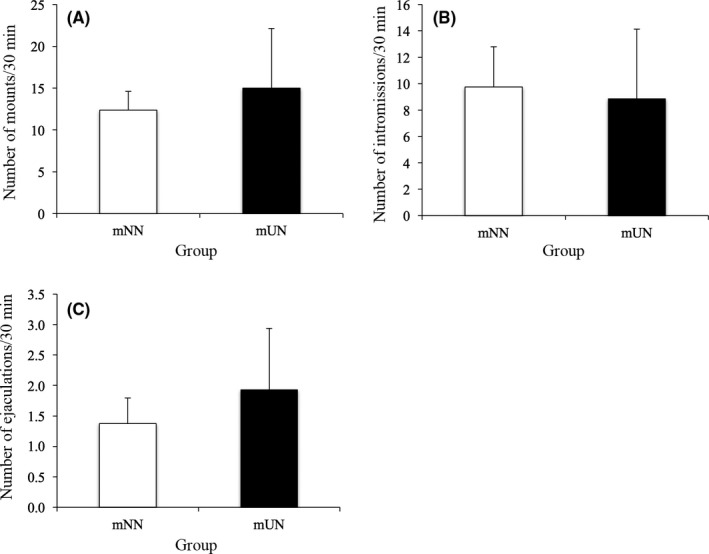
Effect of prenatal undernutrition on sexual behavior. The number of (A) mounts, (B) intermissions, and (C) ejaculations in a 30 min observation did not show a difference between the maternal normal nutrition (mNN) and the maternal undernutrition (mUN) groups. The data are presented as the mean+standard error

The mUN group showed a significantly later day of PS (mNN: 31±0.60 days; mUN: 35.29±1.38 days, *P*<.05) and a heaver body weight on the PS day (mNN: 110.49±3.26 g; mUN: 130.93±7.69 g, *P*<.01), compared to the mNN group (Figure 2). The cumulative PS rate was significantly lower between PND 30‐34 in the mUN group than in the mNN group. The sexual behavior, mounts, intermissions, and ejaculations did not show any difference between groups (Figure 3).

## 4 DISCUSSION

In the present study, it was found that PS was delayed, but that sexual behavior was not modified by prenatal undernutrition in mature male rats. This is the first study to prove the lack of a relationship between prenatal undernutrition and sexual behavior in adulthood in male rats.

The authors have reported that the onset of puberty and vaginal opening of female rats were delayed by prenatal undernutrition in rats.^4^, ^5^ In the present study, PS, which is a reliable finding of puberty, was significantly delayed in the mUN group, compared to the mNN group. Delayed sexual maturation has been studied widely under various types of stressful conditions, including psychological, immune, and metabolic conditions. For example, undernutrition during the prenatal or early neonatal period already has been reported to delay the day of PS in male rats.^6^, ^15^ Additionally, an injection of LPS on PND 3 or PND 5 or atrazine, an agricultural herbicide, on PND 23 also delayed the day of PS in male rats.^9^, ^11^ On the contrary, a low protein diet in the mother during pregnancy and lactation suppressed the sperm count after maturation but did not delay the day of PS in male rats.^16^ The result of the present study confirmed the previous studies regarding the suppressive effect of metabolic stress in the prenatal period on the sexual maturation of male rats. This result further supports the evidence that exposure to stressors in the prenatal and early neonatal periods has long‐term suppressive effects on the sexual maturation of male rats, as well as the metabolic abnormalities in later life that have been reported widely. Furthermore, as shown in Figure 2B, the mUN group required a heavier body weight (i.e. so much growth for PS, compared to the mNN group). The authors previously reported a similar phenomenon in female rats that prenatally undernourished female pups showed a lower expression of Kiss1 mRNA in the hypothalamus during the pubertal period than the normal female pups.^4^ The prenatally undernourished male pups in the present study also might have had lower Kiss1 mRNA expression in the hypothalamus, which could be the reason for the delayed PS in the male pups in the present study.

Furthermore, there are several reports to show that prenatal and early neonatal stressors, either psychological or immune, might suppress sexual activity in male rats. The maternal restraint during gestation days 18‐22 prolonged the latency of the first mount and intromission and suppressed the number of ejaculations in mature Wistar rats.^17^ Maternal separation during the first 10 days after birth resulted in suppressed numbers of mounts and intromissions and a prolonged latency of only ejaculation in mature Wistar rats.^18^ In the mature Sprague–Dawley male rats, maternal separation during PND 2–10 also prolonged the latency of the first mount and intromission and suppressed the number of ejaculations, but not the number of mounts and intromissions.^19^ Moreover, the number of ejaculations was decreased by the injection of LPS (250 μg/kg, intraperitoneally) in the prenatal period, but not other sexual behaviors,^20^ whereas a LPS (50 μg/kg, intraperitoneally) injection in the early neonatal period suppressed only the number of mounts, but not the other sexual behaviors in mature male Wistar rats.^9^ However, sexual behavior, including the number of mounts, intromissions, and ejaculations, did not show any difference between the groups in the present study. Although prenatal and early neonatal stress might have long‐term suppressive effects on sexual behavior in male rats, the effect is not identical among the studies and might be related to the type of stress, induced period, and strain of rat. A difference in the effect of the stressors on sexual behavior between the present study and other studies mainly would be related to the difference in the kind of stress and also might be related to the sensitivity of each stressor regarding the corresponding initial responsive neurons or neuropeptides, such as corticotropin‐releasing hormone in psychological stress, brain inflammatory cytokines in immune stress, and orexigenic or anorexigenic peptides in metabolic stress. Otherwise, it simply might be related to the strength of the stressors used in the studies. If the former hypothesis is supported, the different effects of the stressors might be brought about by epigenetic modification in the initial responsive neurons or neuropeptides of the stressors; if the latter hypothesis is supported, it could be generated from all the steps of the neuronal circuit, from the initial neurons to the operating neurons, of the sexual behaviors. Further study is needed to clarify the mechanism of the effects of stresses on sexual behavior.

In conclusion, prenatal undernutrition had a long‐lasting suppressive effect on puberty, as well as unfavorable influences on metabolism, but not on sexual behavior in male rats.

## DISCLOSURES

*Conflict of interest*: The authors declare no conflict of interest. *Human and Animal Rights*: This article does not contain any study with human participants that has been performed by any of the authors. All institutional and national guidelines for the care and use of laboratory animals were followed. The protocol for the research project was approved by a suitably constituted ethics committee.
